# The chemical damage of sandstone after sulfuric acid-rock reactions with different duration times and its influence on the impact mechanical behaviour

**DOI:** 10.1016/j.heliyon.2023.e22346

**Published:** 2023-11-28

**Authors:** Qinghe Niu, Mingwei Hu, Jiabin He, Bo Zhang, Xuebin Su, Lixin Zhao, Jienan Pan, Zhenzhi Wang, Zhigang Du, Yuebei Wei

**Affiliations:** a Key Laboratory of Roads and Railway Engineering Safety Control (Shijiazhuang Tiedao University), Ministry of Education, Shijiazhuang, 050043, China; b Liaoning Qingchuang High Tech Construction Industrialization Consulting Co., Ltd, Shenyang, 110179, China; c Hebei Technology and Innovation Center on Safe and Efficient Mining of Metal Mines, Shijiazhuang, 050043, China; d Beijing Research Institute of Chemical Engineering and Metallurgy, Beijing 101149, China; e School of Resources & Environment, Henan Polytechnic University, Jiaozuo 454000, China; f School of Civil Engineering, Luoyang Institute of Science and Technology, Luoyang, Henan, 471023, China

**Keywords:** Pore structure, Acid-rock reaction, Hopkinson pressure bar experiment, dynamic mechanical parameters, Failure pattern

## Abstract

The low-permeability characteristic of sandstone-type uranium deposits has become the key geological bottleneck during the in-situ leaching mining, seriously restricting the development and utilization of uranium resources in China. At present, the blasting-enhanced permeability (BEP) and acidizing-enhanced permeability (AEP) are confirmed to be mainstream approaches to enhance the reservoir permeability of low-permeability sandstone-type uranium deposit (LPSUD). To clarify the synergistic effect of BEP and AEP, the acid-rock reaction and dynamic impact experiments were conducted, aiming to study the effect of chemical reactions on pore structure, dynamic mechanical properties and failure pattern of sandstone. Results show that with the increasing acid-rock reaction time, the total pore volume of samples is promoted largely and exhibits obvious chemical damage. The change of pore volume depends on the pore size, the 100–1000 nm and 1000–10000 nm pores are more susceptible to acid-rock reactions. The dynamic peak strength and the dynamic elastic modulus are decreased and the dynamic peak strain and strain rate are increased when lengthening the acid-rock reaction time, whose evolution laws can be fitted by the logistic expression, the linear expression and the exponential expression, respectively. The acid-rock reactions also have an influence on the fracture development of samples after the dynamic impact. The damaged fractures on the end faces of samples grow from the isolated short fracture, the isolated long fracture to the fracture network, and the damaged fractures on the sides of samples develop from the non-penetration fractures, penetration fractures to the multi-branch fractures. This study clarifies the physical and chemical combined damage mechanism, demonstrates the potential of reservoir stimulation by uniting the BEP and the AEP, and provides a theoretical reference for the reservoir stimulation of LPSUD.

## List of abbreviations

BEP: Blasting-enhanced permeability
AEP: Acidizing-enhanced permeability
LPSUD: Low-permeability sandstone-type uranium deposit
IAEA: International Atomic Energy Agency
SHPB: Split Hopkinson pressure bar
ISL: In situ leaching
MIP: Mercury intrusion porosimetry

## 1 Introduction

**Fig. 1:**
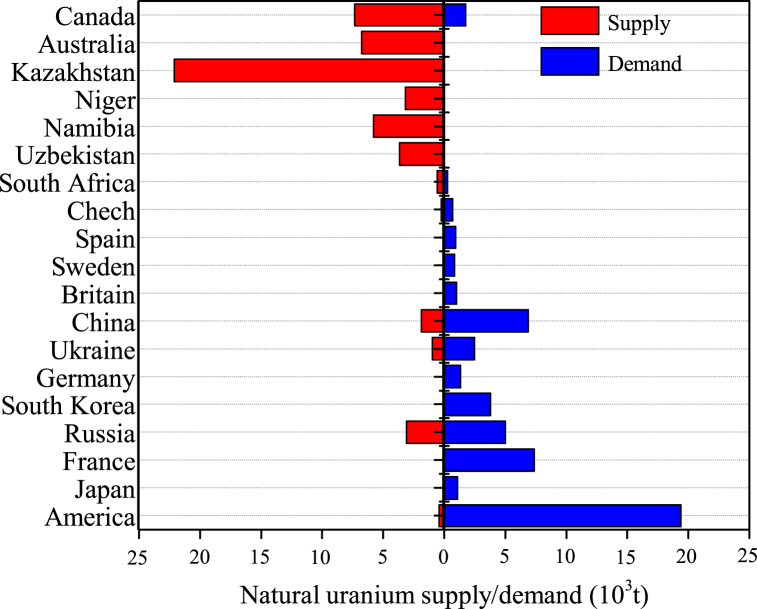
Annual supply and demand of natural uranium in major countries in the world [1].

Natural uranium is the cornerstone of the nuclear military industry and the granary of nuclear power, the demand for uranium resources has increased sharply with the rapid development of the social economy and the continuous optimization of energy structure in China. Statistics from the International Atomic Energy Agency (IAEA) show that the current supply of natural uranium in China only accounts for 1/4 of the demand (Fig. 1) [1]. The proportion of supply and demand of natural uranium is seriously unbalanced, and the dependence on imports remains high [2,3]. Therefore, there is an urgent need to increase the production capacity of natural uranium and the self-sufficiency rate of uranium resources, aiming to ensure energy security and accelerate the construction of a nuclear industry powerhouse [4,5].

**Fig. 2:**
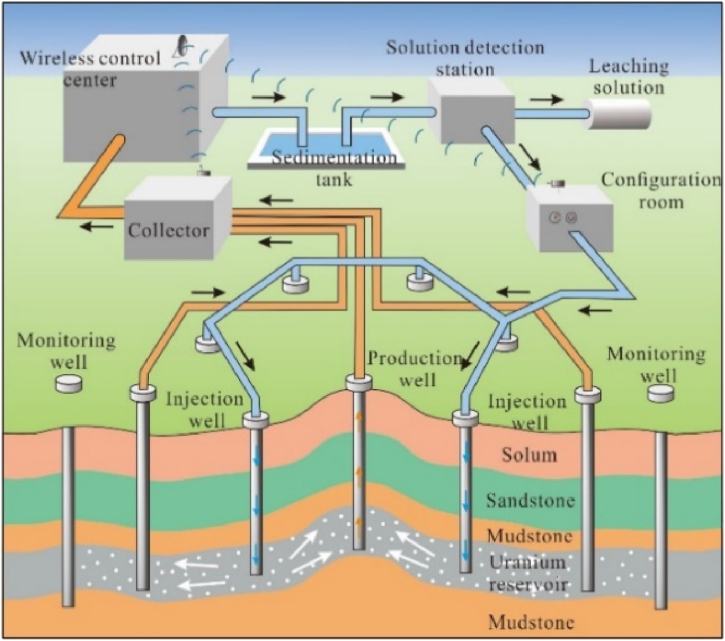
Schematic diagram of *in-situ* leaching mining of sandstone-type uranium deposits [8].

The uranium-bearing strata in China always have a quite low seepage capacity (the permeability is lower than 0.1 m/d). Reports show that the low-permeability sandstone-type uranium deposit (LPSUD) has occupied 70 % of all uranium resources [6]. In China, sandstone-type uranium resources account for the largest proportion [7], and its main development mode is in-situ leaching (ISL) mining. ISL realizes the uranium extraction by injecting a leaching solution into the porous network of the ore body, extracting the uranium through processes of oxidation, dissolution and complexation, and pumping the pregnant leach solution to the surface for hydrometallurgy from the producing wells (Fig. 2) [8]. To attain an excellent and wide-range leaching reaction, the uranium-bearing strata must have a moderate permeability [9]. However, the low-permeability characteristic has become the key geological bottleneck restricting the high-yield and efficient development of LPSUD. The common method to improve the permeability of low-permeability reservoirs is reservoir stimulation, including the hydraulic fracturing method [[10], [11], [12], [13], [14]], pressure-relief method [[15], [16], [17]], chemical stimulation method [[18], [19], [20]], high/low-thermal shock method [21,22] and blasting method [23,24], which have been applied in unconventional oil/gas development fields. For the LPSUD, reservoir stimulation methods of blasting-enhanced permeability (BEP) [25,26] and acidizing-enhanced permeability (AEP) [[27], [28], [29], [30]] have carried out several pilot tests in China and the injection rate of wells has improved by 118% [31].

In terms of BEP, Ye et al. [32] performed hydraulic-controlled blasting of a deep hole by the numerical simulation software and estimated the permeability enhancement effect, confirming that the blasting method can produce damaged fractures and greatly improve the permeability of reservoirs. Yan et al. [33] found that the stress waves induced by the cyclic dynamic impact of a split Hopkinson pressure bar (SHPB) device can improve the porosity and permeability of weakly weathered granites, this transformation for rock can ameliorate the permeability of low-permeability reservoirs and is advantageous to the ISL mining process. Based on the discrete element theory, Yuan et al. [34] found that the decoupled charge coefficient affects the blasting fracturing effect of hole wall and rock mass and obtained the optimal decoupled charge coefficient to get the best BEP effect, then, the interaction mechanism of shock wave and detonation gas on fracture generationand the spacing optimization of double hole blasting [35] were clarified and revealed. Wang et al. [36] established the coupled damage-permeability constitutive model of rock and realized the quantitative evaluation of BEP technology. The preliminary theoretical framework of BEP in LPSUD was hence formed.

In terms of AEP, Liao et al. [31] explored the feasibility of permeability improvement of acid-rock reaction by the indoor plunger experiment and found that the acidic fluid systems (including hydrochloride acid, formic acid and acetic acid) can significantly improve the core permeability with an average percentage increase of 763 %. Han et al. [37] investigated the influence of iron-bearing minerals and iron-free minerals on the reservoir permeability and provides theoretical guidance for the application of AEP. Sun et al. [38] further studied the internal mechanism of acid-rock reaction on the reservoir permeability, the enlarged small and large pores/throats induced by the chemical corrosion contributed to the permeability enhancement of reservoirs. Briefly, the acid flooding can dissolve the active minerals and is capable of improving the permeability of the reservoirs [[39], [40], [41]].

With the increase of mining depth, the sandstone-type uranium deposit may possess a lower seepage capacity, becoming the ultra-low permeability reservoir. Therefore, adopting the composite reservoir stimulation method is becoming the future development direction. It is thus promising to ameliorate the reservoir of LPSUD by combining the BEP and AEP. During the acid-rock reaction process, the mechanical properties of rock must be first changed [[42], [43], [44], [45], [46]] and then the reservoir stimulation effect of BEP can be improved. In this process, the reservoir stimulation effect is controlled by the transformation of fracture propagation law and pattern induced by the loading of dynamic impact [[47], [48], [49], [50], [51]]. However, there are few reports about the combined effects of BEP and AEP technologies, and the interaction mechanism of the acid-rock reaction and dynamic impact action is unclear.

Therefore, this paper first designed the acid-rock reaction and dynamic impact experiments to simulate the AEP and BEP induced by the chemical corrosion and stress wave shock. Then the pore structure, the dynamic mechanical property and the failure pattern are also clarified, and the combined damage mechanism of AEP and BEP is finally revealed. This study aims to comprehensively understand the evolution mechanism of rock dynamic mechanical properties after acid rock reaction from a micro perspective, and revealing the effect of composite reservoir stimulation by physical and chemical methods. This study provides laboratory evidence for the potential applications of BEP and AEP technologies on ISL mining and lay a theoretical foundation for the reservoir stimulation and efficient development of LPSUD.

## 2 Experimental section

### 2.1 Sample collection and description

As one of the important sandstone uranium ISL mining bases, the sandstone ore-bearing bed in China's Ordos Basin is the sampling target location. Samples in this work came from the outcrop sandstone stratum in the southwest of Ordos Basin. The original samples were then prepared into cylindrical samples with a diameter of 50 mm and a length of 30 mm along the vertical bedding plane direction. Both ends were grinded and polished to obtain flat-end faces. To make the samples as comparable as possible and improve the reliability of test results, six samples with similar P-wave velocity (3635 m/s to 3780 m/s) were selected.

The minerals in samples were analyzed using a X-Ray Diffraction (Shimadzu XRD-7000) with Cu Kα source radiation at a scanning rate of 2 min^−1^ from 5° to 70°. After qualitative analysis of the minerals, quantitative analysis was performed using a commercial software package (Siroquant™) [52]. The tese results show that the main components of samples in this work are quartz, kaolinite, feldspar, calcite and debris. The mineral particle size distribution is relatively uniform (0.6 mm–1.0 mm), indicating that these samples are appropriate to be compared and analyzed.

**Fig. 3:**
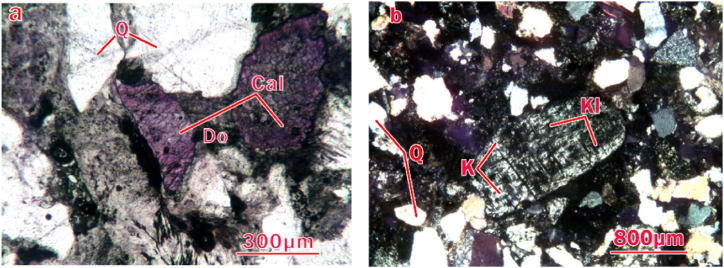
Thin section images of samples (K, Ki, Q, Cal and Do represent potassium feldspar, kaolinite, quartz, calcite and dolomite, respectively).

In addition, the prepared rock samples were ground into thin sheets, then the optical properties, crystallization characteristics and distribution of minerals in samples were determined by a polarization microscope (Fig. 3a and b). It can be seen that the quartz, calcite and potassium feldspar particles are stacked together, forming the main skeleton of rock matrix. The clay minerals (kaolinite and dolomite) are filled in the intergranular pores, which leads to the strong heterogeneity of the internal structure of the samples.

### 2.2 Acid-rock reaction experiment

Considering the acid ratio of ISL field test [53], the sulfuric acid with a concentration of 6 g/L was selected as the acid-rock reaction solution in this work. To ensure that the acid concentration remains constant, the pH of the solution was dynamically measured and the sulfuric acid solution was replenished in time. The reaction time designed in this work was 15 d, 30 d, 45 d, 60 d and 70 d, and a parallel sample without acid-rock reactions was soaked in the deionized water under the same experimental conditions. When the acid-rock reaction experiment was completed, the samples were taken out from the reactor and dried in a temperature control box at 60 °C for 24 h, which were then gathered to conduct other experiments.

### 2.3 Dynamic impact experiment

**Fig. 4:**
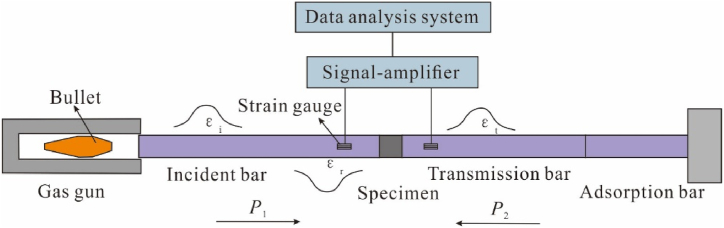
Schematic map of the split Hopkinson pressure bar device designed by Luoyang Liwei Technology Co., Ltd, China and used in Deep Resources Development Laboratory of Shijiazhuang Tiedao University.

The dynamic impact experiment is performed on the SHPB device (Fig. 4), which is composed of five subsystems, i.e., the drive subsystem, the bar subsystem, the energy absorption subsystem, the signal acquisition and data processing subsystems. The length of the bullet, incident bar and transmission bar of the device is 300 mm, 3000 mm and 2000 mm, respectively, and the diameter of them is 50 mm. They are made of 40Cr alloy steel, with a density of 7810 kg/m^3^, a longitudinal wave velocity of 5410 m/s, an elastic modulus of 210 GPa and a Poisson's ratio of 0.23. The driving gas pressure in this work was maintained at 16 psi. The direct loading of impact load will produce a rectangular wave in the bar and cause an obvious dispersion effect in the process of signal propagation [54]. A rubber sheet is used to better realize constant strain rate loading. And an appropriate amount of vaseline was used on the end face of the sample to reduce the friction effect between the sample and the bars.

During the impact process, the voltages measured by the resistance strain gauges pasted on the incident bar and transmitted bar can be used to calculate the incident strain (*ε*_i_), the reflected strain (*ε*_r_) and the transmitted strain (*ε*_t_). According to the one-dimensional stress wave theory, the strain rate ($\dot{\varepsilon }$), the strain (*ε*) and the stress (*σ*) of measured samples were calculated as follow [55,56]:

$$\sigma =\frac{AE}{2A_{0}}\left[\varepsilon_{i}+\varepsilon_{r}+\varepsilon_{t}\right]$$

(1) $$\begin{array}{c} \sigma =-\frac{c}{L_{0}}\int_{0}^{t}\left[\varepsilon_{i}+\varepsilon_{r}-\varepsilon_{t}\right]dt \end{array}$$

$$\dot{\varepsilon }=-\frac{c}{L_{0}}\left[\varepsilon_{i}+\varepsilon_{r}-\varepsilon_{t}\right]$$

where *A* and *E* are the cross-sectional area and the elastic modulus of the pressure bar, respectively. *A*_0_ and *L*_0_ are the cross-sectional area and the length of the sample, respectively. *t* is the duration of stress waves, *c* is the longitudinal wave velocity of the pressure bar, $c=\sqrt{E/\rho }$, *ρ* is the density of the pressure bar.

### 2.4 Pore structure test

A high-pressure mercury intrusion porosimetry (MIP) method was adopted to measure the pore structure of the samples. The injection pressure of mercury ranges from 0 MPa to 228 MPa, the measurement range of pore throat diameter falls between 0.005 μm and 360.000 μm. When increasing the pressure of mercury from small to large in a certain range, the volume of mercury injected into the sample during the pressurization process was recorded. The parameters that characterize the porosity of samples can be obtained from curves between the pressure and the injection volume of mercury. The Washburn equation was used to reflect the relationship between the pore size and the applied pressure during the test process [57]:

(2) $$\begin{array}{c} P\left(r\right)=\frac{-4\gamma \;\cos\;\theta }{d} \end{array}$$

where *P*(*r*) is the mercury injection pressure, MPa; *γ* is the surface tension of mercury, 0.485 J/m^2^; *θ* is the angle of the surface contacting with the solid surface, which equals 130°; *d* is the pore throat diameter, μm. The test process of MIP was based on the International Organization for Standardization (ISO 15901–1:2016).

## 3 Results

### 3.1 Pore structure evolution

**Fig. 5:**
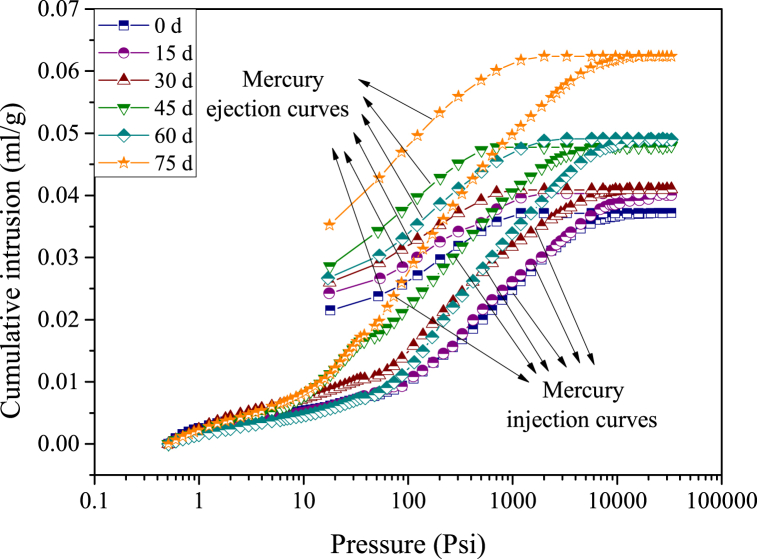
The mercury injection and ejection curves of samples (d - day (24 h)).

The mercury injection and ejection curves of samples at different reaction times are shown in Fig. 5. Generally, the mercury injection and ejection curves are similar, independent of the acid-rock reaction time. The curves of cumulative pore volume show a nonlinear increasing trend with the increasing mercury pressure. This is because the mercury can enter into the pores until the mercury pressure is larger than the capillary pressure, and then the curves of cumulative pore volume reach stabilization when all the connected pores in the sample are full of mercury. However, the cumulative injection volume increases as a function of acid duration, indicating that the pore structure must be changed after the acid-rock reaction experiment.

**Fig. 6:**
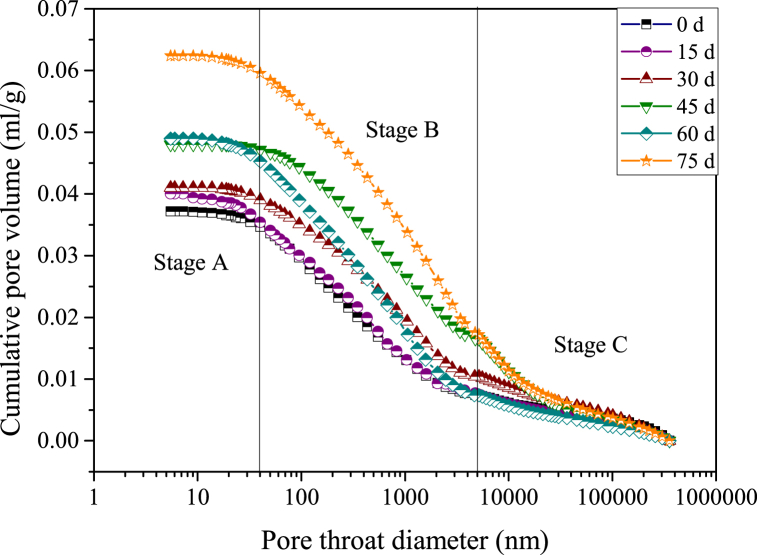
Relationship between the cumulative pore volume and the pore throat diameter (samples are reacted with the acid solution for 0 d, 15 d, 30 d, 45 d, 60 d and 75 d, d - day (24 h)).

**Fig. 7:**
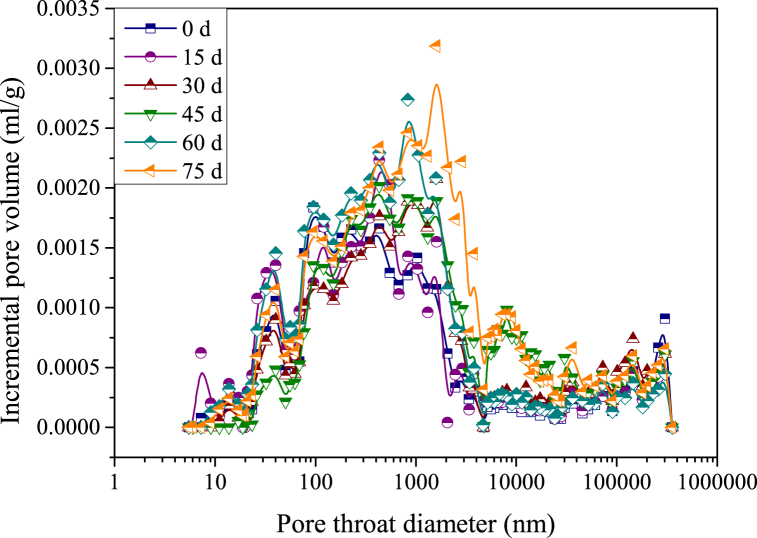
Relationship between the incremental pore volume and the pore throat diameter (samples are reacted with the acid solution for 0 d, 15 d, 30 d, 45 d, 60 d and 75 d, d - day (24 h)).

The relationships between the cumulative pore volume and the pore throat diameter of samples at different acid-rock reaction times are shown in Fig. 6. The pores in samples are multifarious and unevenly distributed in different scales, manifesting in the fact that the cumulative pore volume and the pore throat diameter are nonlinearly correlated. To characterize this evolution relationship, the curves were divided into three stages: stage A, stage B and stage C, which represent the pore size distribution sections of >5000 nm, 20–5000 nm and <20 nm. Compared with stage A and stage C, the cumulative pore volume shows a significant increase phenomenon in stage B. This indicates that the pores with a diameter of 20–5000 nm are massively distributed in the samples, which occupy 66.87–83.41 % of the total pore volume. This can also be authenticated by the relationship between the incremental pore volume and the pore throat diameter (Fig. 7).

**Fig. 8:**
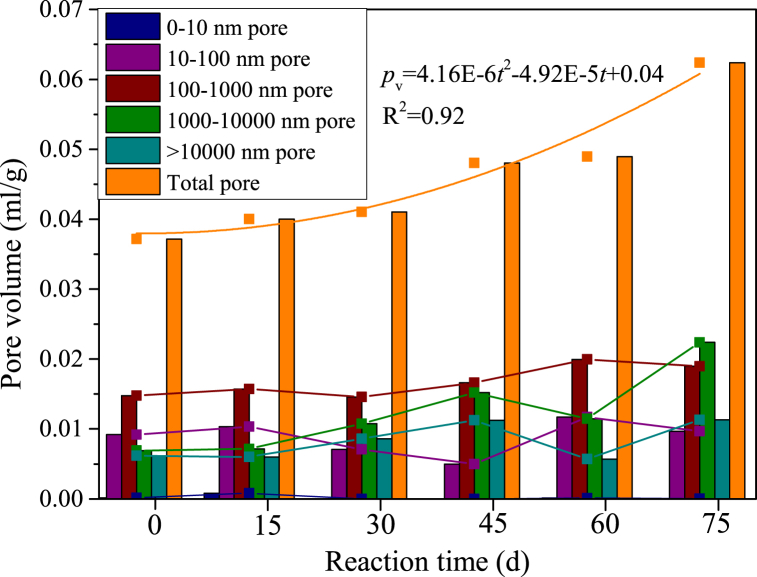
Volume distribution of different size pores in samples (samples are reacted with the acid solution for 0 d, 15 d, 30 d, 45 d, 60 d and 75 d, d - day (24 h)).

The pore volume distribution of samples with various acid-rock reaction times were shown in Fig. 8. The volume of pores with different sizes shows a complex evolutionary relationship, while the total pore volume (*p*_v_) highly depends on the acid-rock reaction. When the reaction times of acid rock were 0 d, 15 d, 30 d, 45 d, 60 d and 75 d, the *p*_v_ (total pore volume) is 0.037 ml/g, 0.040 ml/g, 0.041 ml/g, 0.048 ml/g, 0.049 ml/g and 0.062 ml/g, and the porosity is 9.32 %, 9.69 %, 10.91 %, 11.53 %, 13.57 % and 16.78 %, respectively. The *p*_v_ shows a binomial relationship with the acid-rock reaction time, which reflects the phenomenon that the acid-rock reaction promotes the increase of original pores and the formation of new pores [[58], [59], [60]]. Additionally, the variation trend of pore volume in different pore size segments is inconsistent. The volume of larger pores with diameters of >10,000 nm, 1000–10000 nm and 100–1000 nm are overall increased, while that of smaller pores with diameters of 10–100 nm and <10 nm is overall decreased. This reflects the influence of the complex dissolution and precipitation process of minerals on pore structure.

### 3.2 Dynamic mechanical characteristics

**Fig. 9:**
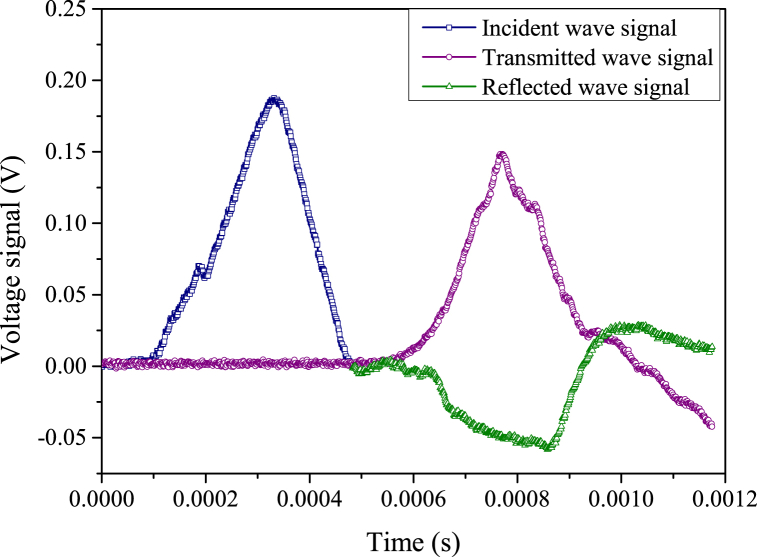
Voltage signals of a typical dynamic impact experiment.

**Fig. 10:**
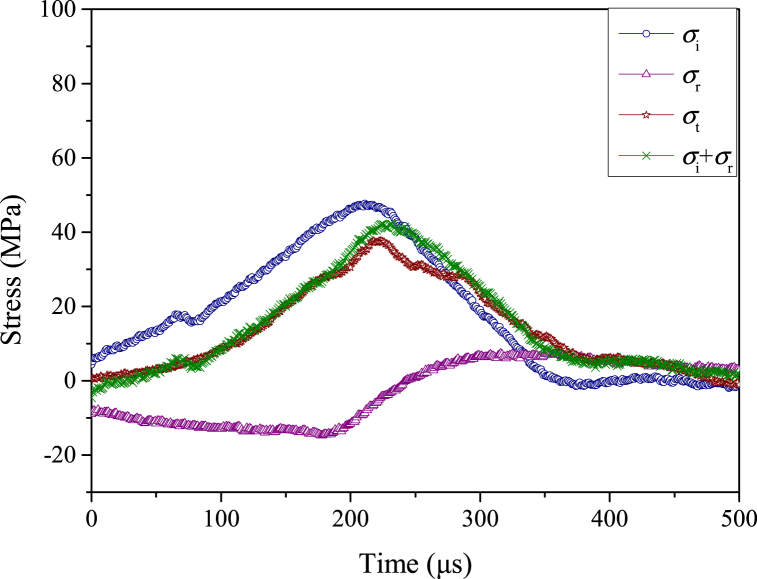
Dynamic stress balance process of dynamic impact experiment. ($\sigma_{i}$**indicates the incident stress,**$\sigma_{r}$**indicates the reflection stress, and**$\sigma_{t}$**represents the transmission stress.**)

The sample with acid-rock reaction time of 45 d is used as an example of dynamic stress equilibrium, the voltage signals and dynamic stress balance process are shown in Fig. 9, Fig. 10, respectively. With the increases in experimental time, the voltage signals of strain gauges during the dynamic impact experiment are continuous and stable, and there is a good platform segment for the reflected wave signal. This means that the test system is stable and the constant strain rate loading mode is reached during the whole test, the test results are thus reliable and convincing [61]. Additionally, the superposition of the incident stress (*σ*_i_) curve and reflected stress (*σ*_r_) curve is close to the transmitted stress (*σ*_t_) curve, indicating that the dynamic stress equilibrium condition is reached and the test is standard and normative. The inertial effect can be ignored in this test because there is no global force difference in the sample to induce inertial forces [62].

**Fig. 11:**
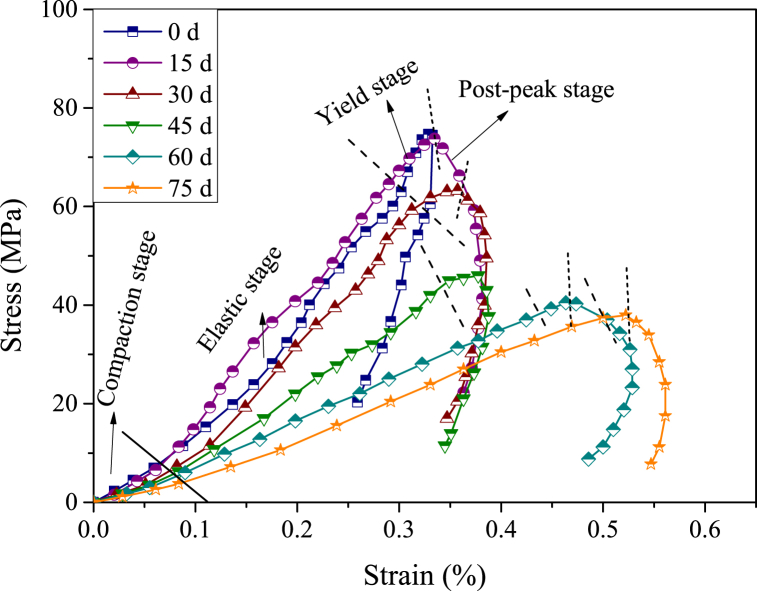
Dynamic stress-strain curves of samples with different acid-rock reaction times (d - day (24 h)).

Dynamic stress-strain curves of samples with different acid-rock reaction times are shown in Fig. 11. Similar to static stress-strain curves, the dynamic stress-strain curves also perform the phased evolution, which can be divided into four stages: the compaction stage, the elastic stage, the yield stage and the post-peak stage. In the compaction stage, the curves increase slowly, indicating that the original pore-fracture structure in samples is first squeezed and the strain is largely advanced even at a lower loading stress. In the elastic stage, the stress-strain curves approach straight lines because the space in samples is compacted and the samples are regarded as elastomers. The yield stages are not obvious because the samples present strong brittleness characteristics, the damaged fractures develop at an extremely high speed to enter into the post-peak stage. In the post-peak stage, the strength of samples reduces instantaneously. The biggest differences in the stress-strain curves are the extension of the compaction stage and the reduction of peak values of samples with the increasing acid-rock reaction time.

To describe the variations of dynamic mechanical properties, the dynamic peak strength (*σ*_p_), the dynamic peak strain (*ε*_p_) and the dynamic elastic modulus (*E*) are calculated. The dynamic elastic modulus of samples was calculated by the method recommended by the International Society for Rock Mechanics (ISRM) [63]:

(3) $$\begin{array}{c} E=\frac{\dot{\sigma }}{\dot{\varepsilon }} \end{array}$$

where $\dot{\sigma }$ is the stress loading rate and equal to the slope of the approximate straight-line rising segment in the stress-time curve; the $\dot{\varepsilon }$ is the strain rate and equal to the average value of the flat stage of the strain rate-time curve.

The results of the dynamic impact experiment for samples with acid-rock reaction times were shown in Table 1. When the acid-rock reaction time varies from 0 d, 15 d, 30 d, 45 d, 60 d–75 d, the *σ*_p_ decreases from 74.48 MPa, 73.75 MPa, 63.25 MPa, 46.00 MPa, 40.50 MPa–37.95 MPa, with a maximum decrease percentage of 49.05 %; the *E* reduces from 26.01 GPa, 20.44 GPa, 18.29 GPa, 12.32 GPa, 7.54 GPa–6.80 GPa, with a maximum decrease percentage of 73.86 %; the *ε*_p_ increase from 0.329 %, 0.335 %, 0.357 %, 0.378 %, 0.473 %–0.522 %, with a maximum increase percentage of 58.66 %; the $\dot{\varepsilon }$ accretes from 18.30 s^−1^, 20.30 s^−1^, 20.60 s^−1^, 22.90 s^−1^, 35.90 s^−1^ to 49.00 s^−1^, with a maximum increase percentage of 167.76 %. The evolution of dynamic mechanical properties is related to the chemical damage induced by acid-rock reactions, which will be discussed in detail in the next section.

**Table 1:** The results of the dynamic impact experiment for samples with different acid-rock reaction times.

Reaction time (d)	*σ*_p_ (MPa)	*E* (GPa)	*ε*_p_ (%)	$\dot{\varepsilon }$ (s^−1^)
0	74.48	26.01	0.329	18.30
15	73.75	20.44	0.335	20.30
30	63.25	18.29	0.357	20.60
45	46.00	12.32	0.378	22.90
60	40.50	7.54	0.473	35.90
75	37.95	6.80	0.522	49.00

## 4 Discussion

### 4.1 Chemical damage induced by the acid-rock reaction

The acid-rock reaction is the immediate cause inducing the deterioration of rock due to the chemical corrosion effects, which has been agreed and demonstrated by many reports [[64], [65], [66], [67], [68]]. To quantify this weakening effect of dynamic mechanical properties, the chemical damage variable (*D*_c_) is defined as:

(4) $$\begin{array}{c} D_{c}=1-\frac{f_{1}}{f_{0}} \end{array}$$

where *f*_1_ and *f*_0_ are the dynamic mechanical parameters after and before the acid-rock reaction. The dynamic mechanical parameters include the maximum strain, elastic modulus and residual strength.

The dynamic mechanical parameters are the quantitative indicators of the mechanical deterioration process, thus, Eq. (4) can not appropriately reflect the chemical damage mechanism and the process of the acid-rock reaction. If the sandstone is extremely dense and no pores occur, the chemical reactions only appear on the surface of samples, however, the pores are developed in samples and the sulfuric acid solution can flow along with the connected pore structure, inducing the fact that the reaction rate is accelerated and the reaction range is enlarged [68]. This can be explained by the research by Ref. [69], who directly observed the dissolution phenomenon of sandstone with sulfuric acid solution from the formation of new pores observed by X-ray computed tomography slices. It is thus appropriate to characterize the chemical damage by the pore volume of samples. According to the previous study [70], the chemical damage variable in this work is defined as:

(5) $$\begin{array}{c} D_{c}=\frac{V_{t}-V_{0}}{1-V_{0}} \end{array}$$

where *V*_0_ is the initial pore volume of the sample before the acid-rock reaction, and *V*_t_ is the pore volume of the sample after the acid-rock reaction of *t* days.

**Fig. 12:**
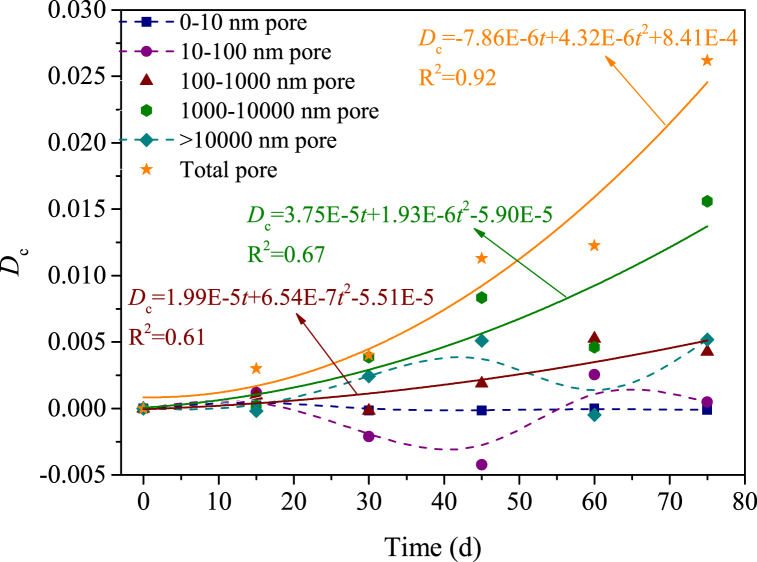
Relationship between the chemical damage variable and the acid-rock reaction time (samples are reacted with the acid solution for 0 d, 15 d, 30 d, 45 d, 60 d and 75 d, d - day (24 h)).

Changes in chemical damage variables at different acid-rock reaction times are shown in Fig. 12. On the whole, the *D*_c_ increases as the extension of the acid-rock reaction time, while this law depends on the pore size, i.e., pores with various sizes contribute differently to the chemical damage of samples. The chemical damage of the total pores has a better binomial relationship with the acid-rock reaction time (R^2^ = 0.92), while that of 100–1000 nm and 1000–10000 nm pores has a moderate binomial relationship with the acid-rock reaction time (R^2^ = 0.61, 0.67); however, there is no obvious relationship between the chemical damage of 0–10 nm pores, 10–100 nm pores and >10,000 nm pores with the acid-rock reaction time. This indicates that the 100–1000 nm and 1000–10000 nm pores dominate the chemical damage during the acid-rock reaction process. Seepage channels composed of small pores are narrow and rugged, which is disadvantageous to the flow of sulfuric acid solution and thus the chemical damage induced by the small pores is inappreciable. The total volume of large pores is minor the chemical damage of these pores is equally weak. The 100–1000 nm and 1000–10000 nm pores have a larger volume and are conducive to seepage of the sulfuric acid solution, which is the dominant pore structure inducing the chemical damage. The other pores have a non-monotonic change trend because of the more complex chemical reactions appear.

### 4.2 Evolution of dynamic mechanical parameters

**Fig. 13:**
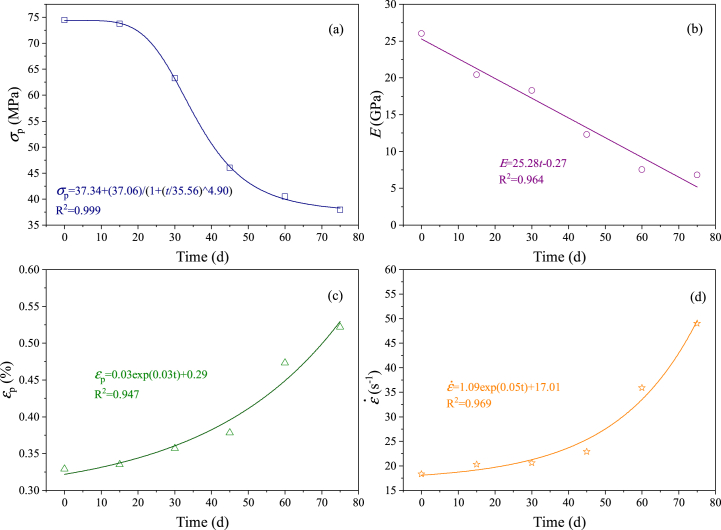
Evolution of dynamic mechanical parameters of samples with increasing acid-rock reaction time (a, b, c and d are the dynamic peak strength, the dynamic elastic modulus, the dynamic peak strain and the strain rate; d - day (24 h)).

The mechanical deterioration of sandstone during the acid-rock reaction process is manifested in the change of dynamic mechanical parameters of samples. The evolution laws of dynamic mechanical parameters of samples at different acid-rock reaction times are shown in Fig. 13. The *σ*_p_, *E*, *ε*_p_ and $\dot{\varepsilon }$ are highly correlated with the acid-rock reaction time. The logistic expression is appropriate to describe the relationship between *σ*_p_ and acid-rock reaction time (Fig. 13a), the linear expression is appropriate to depict the relationship between *E* and acid-rock reaction time (Fig. 13b), and the exponential expression is appropriate to represent the relationship between *ε*_p_ and $\dot{\varepsilon }$ with acid-rock reaction time (Fig. 13c and d). The fitting correlation coefficients are all larger than 0.94.

**Fig. 14:**
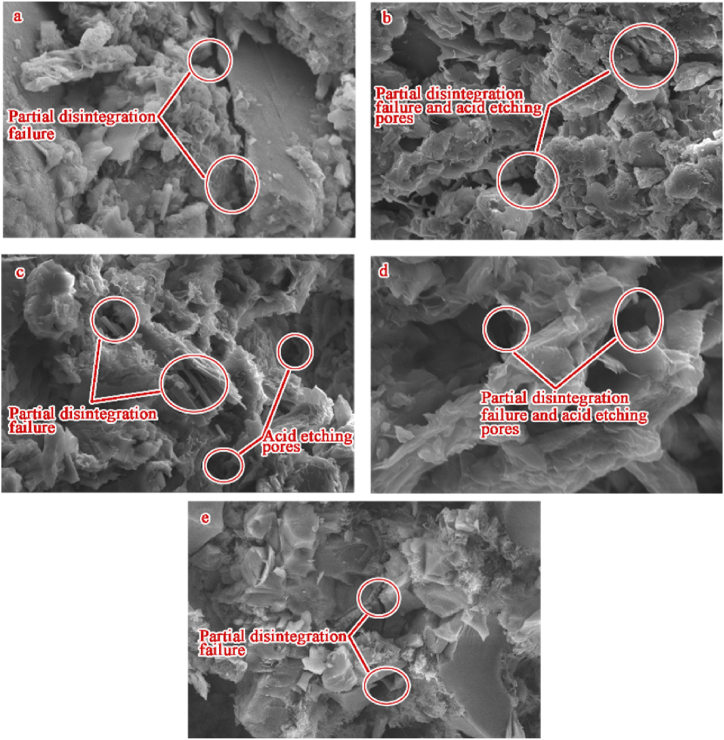
SEM images of the sample treated by the acid-rock reaction after dynamic impact experiment (SEM resolution is between 2 μm and 5 μm).

The *σ*_p_ is phased associated with the acid-rock reaction time. Before the first 15 days, the *σ*_p_ is basically maintained at a high value, after the last 15 days, the *σ*_p_ is gradually stabilized and reached the minimum value. The *σ*_p_ shows a dramatic downward trend during the middle 45 days. The sulfuric acid solution should first dissolve the surface soluble minerals of samples and improve the seepage capacity [54], which creates opportunities for the sulfuric acid solution to further dissolve internal soluble minerals [60]. When the soluble minerals are dissolved completely within a certain range (Fig. 14b,c,d), the samples are deteriorated and easily form damaged fractures after the dynamic impact experiment (Fig. 14a,e), and then the *σ*_p_ reaches the minimum. The *E* shows a monotonically decreasing trend with the acid-rock reaction time, indicating that the acid-rock reaction cannot change the rock material properties and the samples also show an obvious elastic stage though they have been degraded [71]. The *ε*_p_ and $\dot{\varepsilon }$ are increased during the acid-rock reaction process, which shows a “first increases slowly and then increases rapidly” trend. At the same impact speed, the strain and strain rate are increased just because of the degradation of dynamic mechanical properties, inducing the samples can produce great deformation at a low impact speed.

In general, acid rock reaction efficiently dissolves the internal fillers (calcite, etc.) of rock pores, which increases the volume of rock pores, and the continuous dissolution of sulfuric acid solution continues to cause damage to the pores of rock samples, and the degree of damage increases with the increase of time. Therefore, when subjected to dynamic impact, the longer the reaction time, the lower the dynamic mechanical strength, and the greater the deformation.

**Fig. 15:**
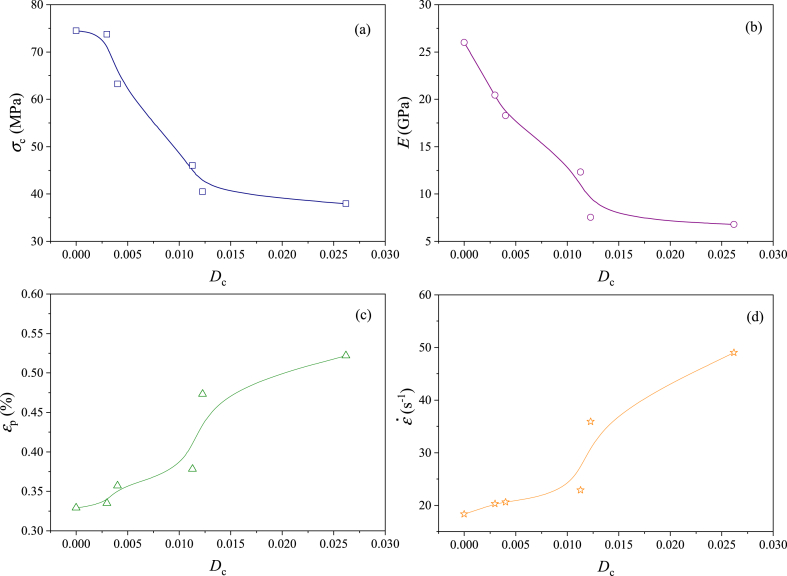
Relationship between the dynamic mechanical parameters and the chemical damage variable (a, b, c and d are the dynamic peak strength, the dynamic elastic modulus, the dynamic peak strain and the strain rate; d - day (24 h)).

The relationships between the dynamic mechanical parameters and the chemical damage variable are shown in Fig. 15. The chemical damage variable is related to the dynamic mechanical parameters, the *σ*_p_ and *E* are negatively related to the chemical damage variable (Fig. 15a and b), which the *ε*_p_ and $\dot{\varepsilon }$ are positively related to the chemical damage variable (Fig. 15c and d). This indicates that the chemical damage variable is correct to describe the dynamic properties and can reflect the mechanical deterioration of the acid-rock reaction on the samples. However, the dynamic mechanical parameters tend to be stable when the *D*_c_ is larger than 0.015. This demonstrates that when the samples are seriously damaged, taking the pore volume to calculate the chemical damage variable may be inaccurate. At this stage, the pore morphology, pore distribution and fractal dimension should be emphasized for causing chemical damage to sandstone [72].

### 4.3 Evolution of failure pattern

**Fig. 16:**
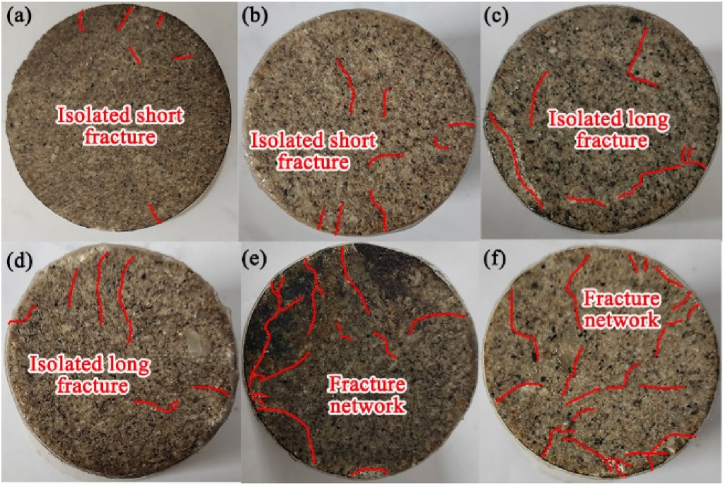
The failure pattern of end faces, (a), (b), (c), (d), (e) and (f) represent the acid-rock reaction time of 0 d, 15 d, 30 d, 45 d, 60 d and 75 d, d means 24 h.

**Fig. 17:**
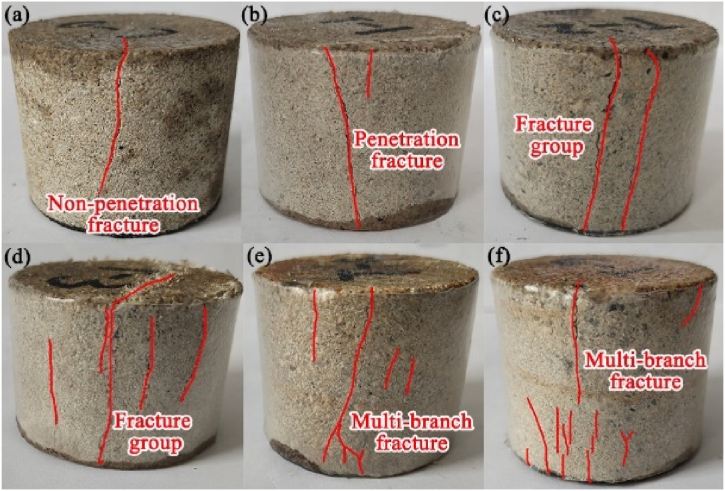
The failure pattern of the sides of samples, (a), (b), (c), (d), (e) and (f) represent the acid-rock reaction time of 0 d, 15 d, 30 d, 45 d, 60 d and 75 d.

The acid-rock reaction affects the pore structure, which thus changes the failure pattern of samples after the dynamic impact experiment. In this work, the failure pattern in the end faces and the sides of samples were investigated, which are shown in Fig. 16, Fig. 17, respectively. The fracture shape and distribution of the samples with different acid-rock reaction times after dynamic impact exhibits large differences. The original sample without acid-rock reaction shows the least damaged fractures, and the fractures are isolated and short, developing on the edge of the sample surface. After a 15 d acid-rock reaction (Fig. 16, Fig. 17b), there are more isolated fractures and they extend to the middle of the sample surface. After 30 d and 45 d acid-rock reactions (Fig. 16b and c and Fig. 17b and c), the isolated short fractures elongate and form the isolated long fractures, however, they are also distributed around the edge of the sample surface. When the acid-rock reaction time reaches to 60 d (Fig. 16, Fig. 17d), the isolated long fractures are connected, and the crisscrossed fracture network is generated. Then after 75 d acid-rock reaction (Fig. 16, Fig. 17e), the coverage area of the fracture network is increased and covers the entire surface of the sample. The damage fracture length statistics are shown in Table 2. With the increase of acid-rock reaction time from 0 d, 15 d, 30 d, 45 d, 60 d–75 d, the total fracture length increases from 46 mm, 70 mm, 125 mm, 149 mm, 174 mm, 213 mm–213 mm, and the connecting fracture length increases from 0 mm, 32 mm, 61 mm, 83 mm, 122 mm–171 mm. This also confirms that the acid-rock reaction changes the internal structure of the sample and subsequently makes it more fragmented after the dynamic impact experiment.

**Table 2:** Damage fracture length statistics.

Reaction time(d)	Total fracture length (mm)	Connecting fracture length (mm)	Number of fractures (pcs)	Fracture density (%)
0	46	0	12	12.22
15	70	32	29	29.56
30	125	61	37	37.70
45	149	83	51	51.97
60	174	122	76	77.45
75	213	171	85	86.62

Similarly, the failure pattern of the sides of samples also depends on the acid-rock reaction. For the original sample without acid-rock reaction, only a non-penetration fracture is developed on the side of the sample, indicating that the original sample has quite high resistance to the impact load. After a 15 d acid-rock reaction, the fracture induced by the impact damage has penetrated the axial direction of the sample and a short-extended fracture is also produced on the edge of the side. After 45 d and 60 d acid-rock reactions, more fractures developed along the axial direction and are combined into a fracture group. With the extension of the acid-rock reaction time, the non-directional fractures are appeared and connected, forming the multi-branch fracture. The complexity of fracture distribution is thus enhanced.

The evolution of the failure pattern shows that the fractures induced by the impact damage are easy to form the interconnected fracture network under the acid-rock reaction, which is advantageous to the reservoir stimulation of low-permeability sandstone uranium deposit. Once the complex fracture networks are generated in the reservoir, the leaching solution can flow along with the complex fracture networks and the overall permeability of the reservoir is promoted, the leaching efficiency will be improved by orders of magnitude. Thus, it can be inferred that combining the BEP and AEP may have a more effective effect on the reservoir stimulation of LPSUD. Nevertheless, it needs more work to quantize the permeability enhancement effect of BEP and AEP, including the establishment of the damage-permeability relationship, permeability attenuation law after reservoir stimulation, the influence of geological condition and construction technology, the field pilot test, which is the follow-up research priorities.

### 4.4 Mechanism of dynamic mechanical deterioration of samples after the acid-rock reaction

This work is organized to investigate the structure property of pores with different sizes and the dynamic mechanical characteristic because they are the direct reflection of the micro and macro of the acid-rock reaction. The sandstones used in this work contain a large number of primary pores and fractures, which provide the space for the chemical reaction and the solution flow [73,74]. The acid solution can migrate from the primary fractures or large pores and react with the soluble mineral, the result is that the pore-fracture volume is advanced and the pore-fracture number is increased, which causes chemical damage to sandstone samples. The pore-fracture structure induced by chemical damage reduces the contacts among mineral particles and instead becomes the initial position of fracture propagation under the effect of dynamic impact [75], this thus leads to the reduction of degradation of dynamic mechanical properties.

The main interactions between sandstone samples and chemical solutions are shown in Table 3. Although the H_2_O can react with the SiO_2_, the reaction rate is very slow and the reaction performance is very weak [76], whose influence on the dynamic mechanical property can be ignored. Another reaction is that the kaolinite can swell after absorbing water [77], which is obvious in our experiments because the leaching solution becomes turbid and a large amount of clay powder remains on the surface of the sample. The influence of water absorption swelling of kaolinite can reduce the hardness of mineral particles and contribute to the decrease of the dynamic mechanical property to a certain extent.

**Table 3:** The main interactions between sandstone samples and chemical solutions [61].

Solutions	Chemical reaction equations
Distilled water	SiO_2_ + H_2_O → H_4_SiO_4_
	CaO + H_2_O → Ca(OH)_2_
	MgO + H_2_O → Mg(OH)_2_
	K_2_O + H_2_O → 2 K^+^ +2OH^−^
H_2_SO_4_ solution	SiO_2_ + H^+^ → Si^4+^ + 2H_2_O
	CaCO_2_ + 2H^+^ → Ca^2+^ + H_2_O + CO_2_↑
	KAlSi_3_O_8_ + 4H^+^ + 4H_2_O → Al^3+^ + K^+^ + 3H_4_SiO_4_
	NaAlSi_3_O_8_ + 4H^+^ + 4H_2_O →Na^3+^ + Al + + 3H_4_SiO_4_
	KAl_3_Si_3_O_10_(OH)_2_ + 10H^+^ → 3Al^3+^ + K^+^ + 3H_4_SiO_4_
	Al_4_[Si_4_O_10_]·(OH)_2_ + 6H^+^ → 4H_2_O + Al^3+^ + 4SiO_2_↓

**Fig. 18:**
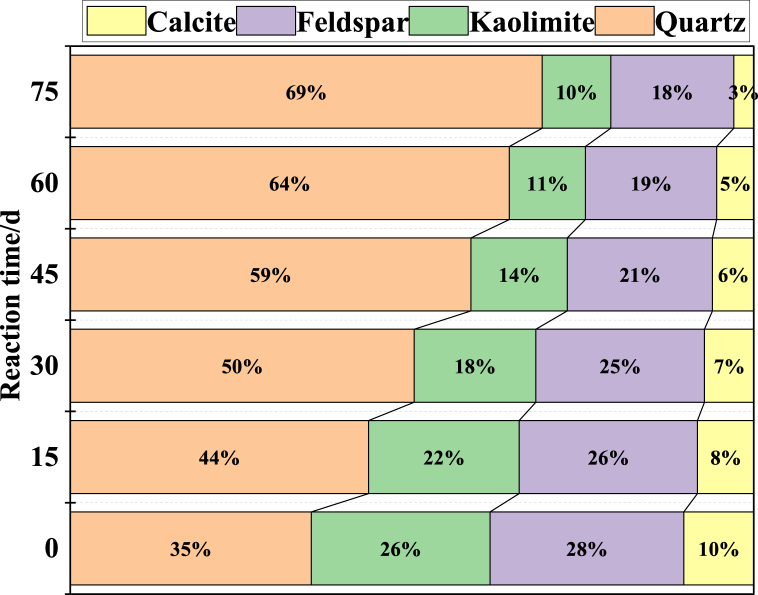
Changes in main minerals of samples during the acid-rock reaction process measured by the X-ray diffraction.

Besides, as shown in Fig. 17, the main minerals of quartz, feldspar, kaolinite and calcite can all react with the H^+^, the products are orthosilicic acid, water, CO_2_ and metal ions. The feldspar, kaolinite and calcite contents are all decreased respectively from 28 %, 26 %, 10 %–18 %, 10 %, 3 %, while the quartz content is increased from 35 % to 39 % (Fig. 18). The dissolution reactions of feldspar, kaolinite and calcite cause chemical damage, and change the pore-fracture structure. The results in 3.1 section show that the increase in pore volume is mainly caused by medium and large pores, indicating that the acid solution can hardly enter the micropores. However, during the *in-situ* leaching process, the leaching solution has high pressure, whether the leaching solution can enter the micropore on a long-time scale needs to be further studied.

This work demonstrates the potential of permeability enhancement effects uniting the reservoir stimulation methods of BEP and AEP. The pre acid-rock reaction of the reservoir can reduce the dynamic mechanical properties, and decreases the critical strength threshold required for fracture propagation and stimulate more fracture networks. The fracture networks induced by the BEP allow more sufficient chemical reactions between acid solution and sandstone. Therefore, the permeability enhancement effect may be more obvious when combining these two methods, which can provide a theoretical reference for the reservoir stimulation of LPSUD.

## 5 Conclusions

(1) The pores with a size of 20–5000 nm are dominated in the sandstone samples, independent of the acid-rock reaction. The total pore volume significantly improves as the acid-rock reaction time increases from 0 to 75 days, with a peak percentage increase of 67.57 %. This indicates that the acid-rock reaction results in substantial chemical damage that cannot be overlooked. The chemical damage variable is positively correlated with the acid-rock reaction time and can be described by the binomial expression. However, pores with various pore sizes are affected differently. The pores with a size of 100–1000 nm and 1000–10000 nm are conducive to the seepage of sulfuric acid solution and are the dominant structure that induces the chemical damage.
(2) As the reaction time of acid rock increases, the rock's interior is progressively dissolved and damaged. This continuous process leads to the development of primary fractures and acid-induced new fractures, which in turn results in a decrease in the rock's dynamic peak strength and dynamic elastic modulus. The rock's internal pore structure suffers significant damage, making it more susceptible to damage under impact load. This leads to a higher degree of fragmentation, and an increase in dynamic peak strain and strain rate. The relationship between dynamic peak intensity and dynamic elastic modulus with acid-rock reaction time can be expressed logically and linearly, respectively. Meanwhile, an exponential expression is suitable for illustrating the relationship between the dynamic peak strain and strain rate with the acid-rock reaction time.
(3) As the reaction time increases, the failure pattern of sandstone post-acid rock reaction remains as brittle failure. The development of fractures following dynamic impact is contingent on the acid-rock reaction, with the pore skeleton strength diminishing over time. The damage fractures on the sample's end face evolved from isolated short fractures, isolated long fractures to the fracture network. Similarly, the damage fractures on the sample's side transitioned from non-penetrating fractures, penetrating fractures to multi-branch faults. This research validates the potential of integrating BEP and AEP for enhancing permeability, offering a theoretical reference for LPSUD reservoir production improvement.

## Data availability statement

Data will be made available on request.

## Additional information

No additional information is available for this paper.

## CRediT authorship contribution statement

**Qinghe Niu:** Conceptualization. **Mingwei Hu:** Writing – review & editing. **Jiabin He:** Writing – original draft. **Bo Zhang:** Methodology. **Xuebin Su:** Software. **Lixin Zhao:** Validation. **Jienan Pan:** Resources. **Zhenzhi Wang:** Formal analysis. **Zhigang Du:** Visualization. **Yuebei Wei:** Validation.

## Declaration of competing interest

The authors declare that they have no known competing financial interests or personal relationships that could have appeared to influence the work reported in this paper.
